# Sleep Patterns and Tryptophan Consumption among Students at Spanish Universities: The Unihcos Project

**DOI:** 10.3390/nu16142376

**Published:** 2024-07-22

**Authors:** María Morales-Suárez-Varela, Carmen Amezcua-Prieto, Isabel Peraita-Costa, Ramona Mateos-Campos, Carlos Ayán, Rocío Ortiz-Moncada, Tania Fernández-Villa

**Affiliations:** 1 Research Group in Social and Nutritional Epidemiology, Pharmacoepidemiology and Public Health, Department of Preventive Medicine and Public Health, Food Sciences, Toxicology and Forensic Medicine, Faculty of Pharmacy and Food Sciences, Universitat de València, Av. Vicent Andrés Estelles s/n, 46100 Burjassot, Spain; isabel.peraita@uv.es; 2 Biomedical Research Center in Epidemiology and Public Health Network (CIBERESP), Carlos III Health Institute, Av. Monforte de Lemos 3-5 Pabellón 11 Planta 0, 28029 Madrid, Spain; carmezcua@ugr.es (C.A.-P.); tferv@unileon.es (T.F.-V.); 3 Department of Preventive Medicine and Public Health, Universidad de Granada, Avenida de la Investigación 11, 18016 Granada, Spain; 4 Instituto de Investigación Biosanitaria ibs.GRANADA, Avda. de Madrid, 15, 18071 Granada, Spain; 5 Area of Preventive Medicine and Public Health, Department of Biomedical and Diagnostic Sciences, Universidad de Salamanca, Calle Alfonso X el Sabio s/n, 37007 Salamanca, Spain; rmateos@usal.es; 6 School of Education and Sports Sciences, HealthyFit Research Group, Universidad de Vigo, Campus A Xunqueira s/n, 36005 Pontevedra, Spain; cayan@uvigo.es; 7 Area of Preventive Medicine and Public Health, Department of Community Nursing, Preventive Medicine and Public Health and History of Science, Food and Nutrition Research Group, Universidad de Alicante, Carretera de San Vicente del Raspeig s/n, San Vicente del Raspeig, 03690 Alicante, Spain; rocio.ortiz@ua.es; 8 Department of Biomedical Sciences, Area of Preventive Medicine and Public Health, Universidad de León, Campus Universitario de Vegazana, 24071 León, Spain; 9 Group of Investigation in Interactions Gene-Environment and Health (GIIGAS), Institute of Biomedicine (IBIOMED), Universidad de León, Campus Universitario de Vegazana, 24071 León, Spain

**Keywords:** students, surveys and questionnaires, tryptophan, Mediterranean diet, cross-sectional study

## Abstract

The objective of this cross-sectional study was to explore sleep patterns and the potential relationship between sleep and tryptophan intake among Spanish university students. A total of 11,485 students self-reported their sleep and dietary patterns and habits. Tryptophan intake was calculated using a food intake matrix and results were presented as quartiles of total intake. Short sleep duration prevalence was 51.0%, with males exhibiting a significantly higher frequency. A total of 55.0% of participants presented inadequate sleep efficiency, with males again presenting a higher rate. Median tryptophan intake was 692.16 ± 246.61 mg/day, 731.84 ± 246.86 mg/day in males and 677.24 ± 244.87 mg/day in females (*p* = 0.001). Dietary tryptophan intake below the first quartile (<526.43 mg/day) was associated with a higher risk of short sleep duration in males (1.26; 95%CI: 1.02–1.55) and females (1.19; 95%CI: 1.05–1.34) and with the Athens Insomnia Scale insomnia in males (2.56; 95%CI: 1.36–4.82) and females (1.47; 95%CI: 1.10–2.05). Regarding academic specializations, females in the humanities field showed a higher risk of Athens Insomnia Scale insomnia due to low tryptophan intake (Q1: 3.15; 95% CI: 1.04–9.55 and Q2: 3.41; 95%CI: 1.01–11.5). In summary, lower tryptophan consumption appears to be associated with poorer sleep quality in Spanish university students; however, other social factors affecting students may also influence sleep quality. These findings have important implications for nutritional recommendations aimed at enhancing tryptophan intake to improve sleep quality.

## 1. Introduction

Sleep deprivation, whether due to behavioral factors or sleep disorders, is becoming increasingly prevalent in modern society [1]. Sleep is one of the pillars of physical, mental, and emotional health and wellbeing, and it is involved in the regulation of various biological processes pivotal to health [2] such as concentration, emotional stability, memory consolidation, immune system regulation, and energy restoration [3]. Short sleep duration has been previously linked with obesity, hypertension, and impaired glucose metabolism [1,4]. Sleep is a biological necessity, and insufficient or poor-quality sleep can have severe consequences for overall health, quality of life, and safety [5].

The prevalence of insomnia symptoms in the general adult Spanish population has been estimated to be 43.4%, the prevalence of chronic insomnia syndrome 13.7%, and chronic insomnia disorder 14.0% (women 14.6%, men 13.4%; 18–34 y.o. 11.1%, 35–54 y.o. 11.5%, 55 + y.o. 17.9%) [6]. In a study on Spanish university students, 39.7% had significant insomnia complaints [7]. The prevalence of chronic insomnia disorder in Spain has more than doubled in 20 years, with an increase of almost 47%, highlighting its significance [6]. 

Sleep problems among young adults are increasing [8,9,10], and specifically among university students, studies have shown a decline in the average number of hours of sleep [11]. Young adults in university experience a challenging transition period in their living arrangements, social life, financial situation, and academic/professional demands marked by greater personal autonomy. It is well documented that university students sacrifice sleep to study and socialize during the week, then sleep long hours on weekends, while alcohol and caffeine consumption and the use of recreational drugs are also commonplace [11,12,13,14,15,16]. Previous studies have reported rates of up to 75% of university students declaring to have occasional sleep problems [17,18]. Taking all of this into consideration, it is not surprising that sleep problems are a common occurrence in university students.

Lack of sleep has been associated with impaired concentration, reduction in academic performance, impaired driving, risk-taking behavior, anxiety, depression, emotional instability, impaired memory, compromised perception of effort and driving performance, impaired social relationships, and poorer health [5,12,13,14,19,20,21,22,23,24,25,26,27,28]. In summary, sleep problems can impair university students’ lives significantly.

Most of the etiological and physiopathological models proposed to understand sleep problems are based on the “3P” model of predisposing, precipitating, and perpetuating factors [29]. Among these factors that can play a role in sleep problems are medical, psychiatric, environmental, and behavioral conditions [30]. Diet would be included as one of these conditions that may be involved in the development of sleep problems. The recent scientific literature has provided interesting insights on the potential association between diet and sleep [31,32,33,34,35]. Diet may influence sleep via melatonin and its biosynthesis from tryptophan [36].

Numerous population studies conducted across various geographical locations and with diverse subject populations have demonstrated that tryptophan intake influences sleep duration by affecting the hypnotic neurotransmitters serotonin and melatonin [37,38]. Tryptophan is an essential amino acid and a precursor of serotonin and melatonin, found mostly in animal products, such as beef, lamb, pork, poultry, and dairy, as well as in nuts and seeds, whole grains, and legumes, which are directly related to sleep quality and duration. The reference dietary intake for tryptophan is 4.5 mg per kilogram of body weight [39,40,41,42]. Therefore, a person weighing 70 kg should consume around 315 mg of tryptophan per day. According to the USDA Food Data Central Repository, beef has an estimated 374 mg of tryptophan per 100 g, lamb has 412 mg/100 g, pork has 369 mg/100 g, poultry 404 mg/100 g, eggs 153 mg/100 g, dairy (milk) 43 mg/100 g, dairy (yogurt) 33 mg/100 g, nuts and seeds (squash and pumpkin seeds) 576 mg/100 g, whole grains (oatmeal) 40 mg/100 g, whole grains (pasta) 45 mg/100 g, whole grains (rice) 30 mg/100 g, legumes (black beans) 105 mg/100 g, legumes (lentils) 80 mg/100 g, and legumes (edamame) 242 mg/100 g.

Melatonin is the main regulator of sleep–wake cycles and serotonin is associated with sleep quality [43]. Studies have previously investigated the association between tryptophan consumption and sleep quality and have shown a relationship between tryptophan consumption and improved sleep [44,45,46]. These studies suggest that supplemental doses as low as 1 g can notably decrease sleep latency and enhance subjective ratings of sleepiness in individuals with insomnia [37,38]. Tryptophan-rich foods have been shown to improve sleep [47,48], while low tryptophan consumption has also been associated with other negative health outcomes, apart from poor sleep, such as depression [49], brain injury [50], cancer, neurodegenerative diseases [51], and autoimmune diseases [52].

However, to the authors’ knowledge, no studies conducted in Spain have ever investigated the relationship between tryptophan consumption and sleep quality in university students. Most of the research up until now has focused on the link between diet and sleep quality on middle-aged or elderly adults but university students find themselves in a crucial stage for the acquisition and consolidation of dietary and sleep-related behaviors that could be an important point of intervention if needed. Thus, this study aimed to evaluate sleep and its possible relationship with tryptophan intake in first-year university students within the uniHcos Project.

## 2. Material and Methods

### 2.1. Design and Ethical Considerations

This cross-sectional study is framed within the uniHcos project, a multicenter study of multipurpose prospective cohorts involving 11 Spanish universities (León, Vigo, Jaén, Granada, Salamanca, Huelva, Alicante, Cantabria, Valladolid, Castilla-La Mancha, and Valencia) [53], whose general objective is to understand the lifestyle habits with which students enter university and their modification during this time period. 

The UniHcos project obtained approval from the Ethics Committees of collaborating universities, and the integration of information files adhered to European and Spanish laws concerning personal data protection. This study was conducted in accordance with the principles of the Declaration of Helsinki. Survey protocols, instruments, and procedures for obtaining informed consent were approved by all participating universities based on these ethical guidelines. 

**Figure 1:**
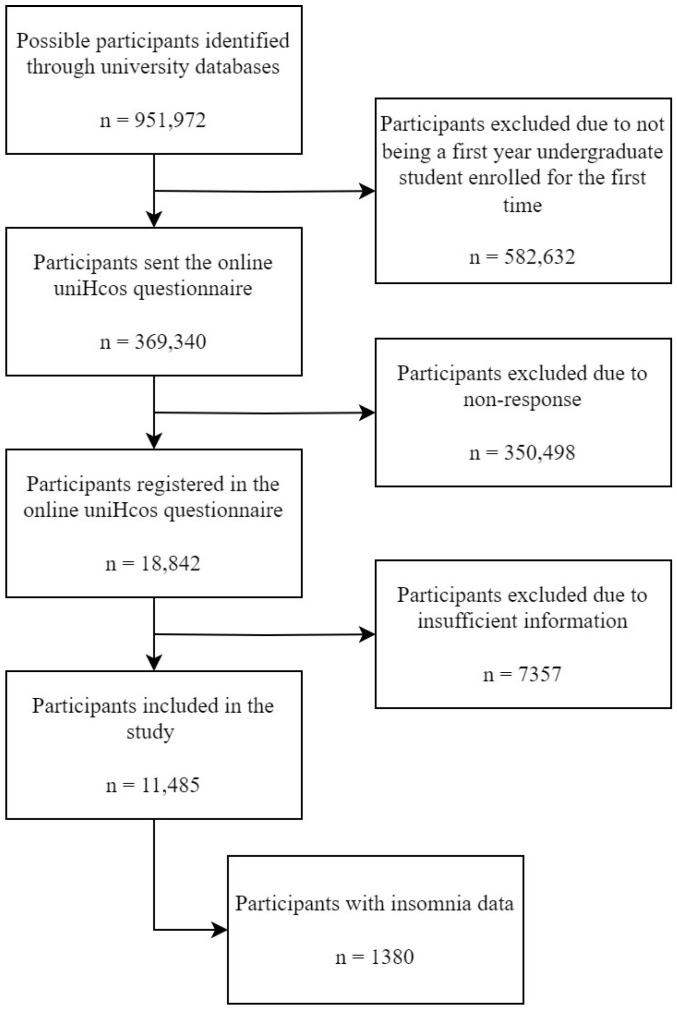
PRISMA flow chart: recruitment of study participants.

The inclusion criteria were to be a first-year student enrolled for the first time in an undergraduate course in one of the participating universities during the 2011–2022 academic years. Students that had been enrolled previously in any undergraduate course, master’s students, and/or doctoral students were excluded. The information needed to assess eligibility (enrollment history) and contact the students (institutional email) was obtained through the secretariats of the participating universities, broken down by campus, center, and degree program. The invitation to participate in the study, including a letter explaining the study, its objectives, and clarifying that the data provided would be treated confidentially, as well as an informed consent form, was sent to all students who met the inclusion criteria through their institutional e-mails. A flowchart detailing the recruitment process is included (Figure 1).

### 2.2. Questionnaire

The surveys were carried out by means of an ad hoc self-administered online questionnaire. A list of the sections that make up the questionnaire used in uniHcos and the surveys on which they are based can be found in previous publications [53]. The SphinxOnline^®^ platform, which allows for the creation of two independent files (personal data on the one hand and the questionnaire variables on the other) coded in such a way that each survey cannot be related to the individual surveyed was used. The program assigns a random code to each subject, which makes it possible to resend to students who have not completed the survey or who have left any part blank, in order to avoid a loss of information, as well as to keep track of those who have participated. Informed consent and ethical permission are also included in the online questionnaire. 

### 2.3. Data Collected

The sociodemographic variables collected were the following: sex (male, female); age (years); marital status (single, domestic partner, married, separated, divorced, or widowed); employment status (only study and I do not look for work, study and I look for work, study and work part-time, study and work full-time); housing, defined as the place where students live during the course (home-family, residence-hall/residence-university, rental, home-own, others); coexistence, defined as people with whom the student lives during the course (with my parents, roommates/friends, with my partner, with my children, alone); and academic specialization (humanities, science, medical, management, engineering, and architecture).

For a better interpretation of the data, some sociodemographic variables were re-categorized:

Marital status: single (single, separated, divorced, widowed), married (married, domestic partner);
Employment status: unemployed (only study and do not look for work, study and look for work), employed (study and work part time, study and work full time);
Housing: family home, university residence (residence hall/university residence), rental (rental, home-own, others);
Coexistence: parents, roommates (roommates/friends), partner (with my partner, with my children), alone.

Sleep-related variables were also collected:

Sleep duration (“Could you tell me, approximately, how many hours you sleep?”);
Short sleep duration (less than 7 h) [54];
Sleep efficiency (“Do the hours you sleep allow you to get enough rest?” yes, no);
Difficulty to sleep (never, some days, several days, most days, every day);
Wake up several times while sleeping (never, some days, several days, most days, every day);
Wake up early (never, some days, several days, most days, every day); insomnia (“Do you suffer from insomnia?” yes, no);
Athens Insomnia Scale [55,56] (no problem, slightly delayed, markedly delayed, very delayed or did not sleep at all, total score: 0–24).

### 2.4. Tryptophan Intake

The tryptophan intake variable was constructed from the answers to the food frequency consumption section (FFCS) of the online self-questionnaire completed by all students modeled after question 96 of Section H4 of the 2006 Spanish National Health Survey and which has been previously validated for the Spanish population. The FFCS has 5 consumption frequency options (daily, 3–4 times/week but non-daily, 1–2 times/week, <1 time/week, never/almost never) for 12 different food categories (fresh fruit, meat, eggs, fish, pasta/rice/potatoes, bread/cereals, vegetables, legumes, charcuterie, dairy, sweets, and sugary drinks). 

Tryptophan intakes were estimated as an average of daily intake by linking the FFCS data on type and weekly frequency of foods consumed to an ad hoc food composition database using information from the U.S. Department of Agriculture, Agricultural Research Service, Beltsville Human Nutrition Research Center. FoodData Central is available from https://fdc.nal.usda.gov/ (accessed on 26 March 2024) thereby creating an exposure matrix for tryptophan intake. Intake was then stratified into quartiles for analysis. To evaluate tryptophan intake adequacy, the estimated daily recommended intake was calculated using the recommendation of 4.5 mg/kg of bodyweight [39,40,41,42]. 

### 2.5. Statistical Analysis

All results are presented for the sample as a whole and stratified by gender. Normality was assessed using the Shapiro–Wilk test. Student’s *t*-test or the Mann–Whitney U-test were used for the comparison between two groups of parametric or nonparametric variable means, respectively. ANOVA or Kruskal–Wallis tests were used for the comparison between 3 or more groups of parametric or nonparametric variable means, respectively. For the comparison between medians, a median test or an expanded median test as a non-parametric test was used. Statistical significance was established at the level of *p* < 0.05 to determine if there were differences between the groups.

Sleep-related factors were analyzed using binary logistic regression (results presented as odds ratios (ORs)) techniques and 95% confidence intervals (CIs) were calculated, stratifying by the tryptophan intake quartile. All results are presented for the sample as a whole and stratified by gender. Student academic specialization was also analyzed using binary logistic regression and a 95% CI and stratifying by the tryptophan intake quartile. All results are presented for the sample as a whole and stratified by gender. 

The analysis of the data was performed with the statistical package IBM-SPSS version 28.0 (IBM Corp. Released 2021. IBM SPSS Statistics for Windows, Version 28.0., Armonk, NY, USA).

## 3. Results

A total of 11,485 first-year university students were included in the study. Within the characteristics of the study population, women represented 72.7% of the university population studied, with an average age of 20.11 ± 4.62 years, presenting significant differences between the genders, with 20.47 ± 5.17 years for males and 19.97 ± 4.38 for females.

Table 1 describes the basic sociodemographic characteristics of the population studied according to gender. The female students that participated in this study were around 6 months younger than their male counterparts. Differences were observed between the genders in housing and coexistence during the academic year. Male students were more likely to live at the family home or their own home while female students were more likely to live in private rentals. Along this same line, male students were more likely to live with their parents or alone while female students were more likely to live with roommates. Differences were also observed in employment rate, with male students more likely to work. The biggest differences were observed in the academic field of study of the students, with males more likely to study science, engineering, and architecture and females more likely to study humanities, medical, and management courses.

Table 2a,b show the results for the sleep-related variables according to gender. In Table 2a, the results presented are those obtained from specific questions in the uniHcos questionnaire, while Table 2b presents the results specific to insomnia that were obtained from an additional question on the uniHcos questionnaire, and the Athens Insomnia Scale questionnaire completed by 1380 students. Significant differences are observed for all the sleep-related variables presented in Table 2a. While female students report longer sleep duration, they present worse values for all other sleep quality variables. This trend is also reflected in the insomnia-related results presented in Table 2b where female students self-report significantly higher rates of insomnia and record significantly worse Athens Insomnia Scale scores. 

Table 3 shows the calculated tryptophan requirement, tryptophan intake, and the ratio between the two values. The values for the tryptophan intake quartiles determined were Q1 (<526.43 mg/day); Q2 (526.43–663.65 mg/day); Q3 (663.65–841.60 mg/day); and Q4 (>841.60 mg/day). The estimated tryptophan requirement is bodyweight-dependent and therefore significant differences between the genders are expected. Males presented a median requirement over 20% greater than female students; however, their median intake was only around 8% higher. Therefore, the calculated intake/requirement ratio was significantly higher in female students. When the sample is divided by tryptophan intake quartiles, female students predominate in the lowest quartile while male students do so in the third and fourth quartiles, with no differences found in the second quartile.

Table 4 shows the results of the binary logistic regression for the association between some of the sleep-related variables and tryptophan intake quartile. The sleep-related variables presented are short sleep duration, sleep efficiency, insomnia, and Athens Insomnia Scale score. Taking the whole sample of students, those within the lowest tryptophan intake quartile presented an elevated risk of short sleep duration, insomnia, and AIS insomnia while also presenting a decreased risk of adequate sleep efficiency. In female students, the same is observed for the risk of short sleep duration, insomnia, and AIS insomnia but no sleep efficiency. In male students, the elevated risk for short sleep duration and AIS insomnia is maintained. In the total sample, it must be noted that the risk of short sleep duration is also elevated in the third quartile of tryptophan intake, which when separating by gender, is only present in female students.

Table 5 shows the results of the binary logistic regression for the risk of AIS insomnia depending on tryptophan intake quartile and student academic specialization. Taking the whole sample of students, those with humanities, medical, and management specializations within the lowest tryptophan intake quartile presented an elevated risk of AIS insomnia. Humanities students also presented elevated risks of AIS insomnia within the other tryptophan intake quartiles studied. When separating by gender, this elevated risk only appears in female humanities students within the two lowest quartiles of tryptophan intake.

## 4. Discussion

In this extensive university student study, we identified that more than 50% of participants experience sleep problems, with differences noted by gender, and dietary tryptophan consumption exceeded the current recommended levels [39,41,42]. This prevalence of sleep problems in university students is higher than that described in other countries [57,58]. 

Over half of the students surveyed reported short sleep duration (51.0%) or inadequate sleep efficiency (55.0%), which is comparable to the results from other recent studies in Spanish university students [7,59,60,61,62].

Tryptophan consumption was high with median intakes being over twice the estimated daily requirement for both male (2.27 times) and female (2.60 times) students. While global studies focused on dietary tryptophan consumption are limited, previous studies have also shown intake levels in adult populations well over the estimated requirements [63,64,65].

However, we also discovered that participants in the highest quartiles of dietary tryptophan intake had a reduced risk of sleep disorders compared to those in the lowest quartile. Furthermore, dietary tryptophan was positively associated with sleep duration, sleep efficiency, and lower levels of insomnia (Athens Insomnia Scale). Sleep duration partially mediated the relationship between tryptophan intake and sleep quality in Spanish university students. To our knowledge, this is the first study to investigate the impact of dietary tryptophan intake on sleep, as well as the mediating role of sleep duration. Our findings underscore the importance of moderately increasing tryptophan consumption to enhance sleep quality, particularly given that student intake exceeds current recommendations.

Our study’s analysis of the association between tryptophan and sleep duration suggests that tryptophan consumption may moderately increase sleep time under real-life conditions. Recent randomized controlled trial studies by Sutanto et al. have evaluated the impacts of 5-hydroxytryptophan (5-HTP) supplementation on sleep quality and the composition of the intestinal microbiota in older adults [66,67]. In these studies, 5-HTP supplementation is shown to improve certain sleep quality components and gut microbiota composition in older adults and this benefit is more prominently observed in poor sleepers [66,67]. While their study population is very different to ours, their results are important given that the quality of sleep gradually decreases during the aging process, and if 5-HTP supplementation can potentially act to restore imbalances in the composition of the intestinal microbiota, it might mitigate cognitive impairments caused by sleep deprivation [68]. Furthermore, support for the beneficial effects of dietary tryptophan on sleep parameters is reinforced by a meta-analysis encompassing 18 studies from diverse ethnicities and regions, which highlighted that tryptophan could enhance various aspects of sleep [37]. 

The present results revealed that students consuming the lowest level of tryptophan had worse sleep outcomes, and coincidently, female students that had lower intakes than their male counterparts presented higher risks for sleep problems. Regarding student academic specialization, humanities students presented a significantly higher risk of sleep problems for all tryptophan intake levels, but when separated by sex, the risk only remained in the two lowest quartiles of female students. It remains unclear if academic orientation plays a significant role in the relation between tryptophan intake and sleep problems. However, this study showed an association between lower tryptophan consumption and sleep problems, even though intake was well above recommendations. This may indicate that the diet as a whole is of a lower quality as the fact that tryptophan requirements are sufficiently covered makes it unlikely that this would be the only reason for poorer sleep.

While comprehensive international studies estimating dietary tryptophan intake are limited [44,45,46,69,70], previous studies, in agreement with the results presented here, confirm the positive association of tryptophan intake and better sleep outcomes [71,72,73]. Furthermore, low tryptophan has been shown to raise the risk of metabolic risk factors and all-cause mortality [49,74].

As an essential amino acid, tryptophan cannot be produced by the human body and must be obtained from food. Tryptophan is essential in several physiological activities, regulating neurobehavioral effects such as appetite, sleeping–waking rhythm, and pain perception [75,76]. It is a precursor in the synthesis of the neurotransmitter serotonin that affects mood, appetite, and sleep and the epiphyseal hormone secreted in the brain, melatonin, which plays a crucial role in regulating the circadian rhythm [75,76]. 

Animal studies have elucidated the potential mechanisms behind these effects. Ardiansyah et al. proposed that both a single oral dose and ongoing treatment with tryptophan improved glucose metabolism and blood pressure in rats, with these benefits mediated by serotonin levels [77]. Tryptophan serves as the substrate for serotonin synthesis and is converted into 5-hydroxytryptophan (5-HT) by tryptophan hydroxylase, which is the rate-limiting enzyme in serotonin production [78]. Tryptophan hydroxylase operates at 50% saturation with its substrate under normal physiological conditions. Therefore, any change in tryptophan availability in the brain can significantly impact serotonin synthesis in the brain [79]. 

Manipulating dietary tryptophan affects plasma tryptophan levels and the ratio of tryptophan to large neutral amino acids (LNAAs). This ratio is critical because it determines the availability of tryptophan across the blood–brain barrier [38]. Serotonin-containing neurons are primarily located in the raphé nuclei of the brainstem and project widely to the cerebral cortex, hypothalamus, and major autonomic nuclei. This extensive network suggests that these neurons exert broad regulatory control over various physiological and behavioral functions [80]. Therefore, it is logical to conclude that dietary tryptophan influences serotonin levels, thereby potentially reducing the risk of sleep problems.

However, the precise mechanism underlying the sedative effect of tryptophan has not been fully elucidated. Tryptophan serves as a precursor for the neurotransmitter serotonin, which in turn is involved in the synthesis of melatonin. Both serotonin and melatonin are believed to play roles in the regulation of sleep and circadian rhythms, and they are used in the treatment of insomnia [63]. 

Moreover, previous studies in humans and experimental animals have convincingly demonstrated the capacity of dietary tryptophan to influence serotonin and melatonin secretion [81]. One of the physiological effects of melatonin is to stimulate peripheral vasodilation, which in turn decreases core body temperature. This reduction in central temperature helps facilitate the onset of sleep [82]. However, like serotonin (5-HT), the precise mechanisms and specific brain areas where serotonin and melatonin act to induce sleep remain unclear. Therefore, further research into these mechanisms is essential for a better understanding of sleep regulation.

Given this, sleep problems due to insufficient dietary tryptophan intake should not be present, but this should be further investigated in a study focused specifically on compliance with tryptophan intake recommendations and its association with sleep problems that also considers other common causes as confounders. However, while in this study causality cannot be attributed, an association with tryptophan intake level and sleep problems is present. Students in the lowest quartiles of intake have a higher risk of sleep problems than those with higher intakes even though they mainly comply with the higher end of the commonly accepted recommendations for tryptophan intake of 4.5 mg/kg of bodyweight [39,40,41,42]. 

## 5. Limitations

This study has certain limitations that must be considered. First, the sample is unevenly distributed according to gender. Almost three-quarters of the participants are female, which in any case is in line with participation rates observed in similar studies. In the 11 participating universities, the average ratio of female students in 2010–2024 is 56.7%, 16% points lower than that of the survey respondents. This is in line with what is known about gender-based survey participation as women tend to be more likely to self-select to participate in online surveys [83]. While acknowledging this participation bias, we do not believe it significantly affects the results of our study. Given that the online questionnaire is very comprehensive and covers many different areas, we do not believe that those with sleep issues would be more or less inclined to respond. However, we do recognize that certain individuals or groups, such as habitual drug or alcohol users, may participate less even when guaranteed anonymity due to social desirability bias. Also, given that the estimated tryptophan requirement is only bodyweight-dependent and individualized, significant differences between the genders are expected and any bias resulting from non-response of certain subgroups of individuals should not affect the results. Therefore, while certain individuals or subgroups of each gender may in fact be more inclined to participate in the survey, the results relating to sleep and tryptophan intake are not expected to be affected. However, ideally, in in order to most accurately assess any gender-based differences, it would be best if the number of male students included in the study could be increased to match that of the female students. 

Regarding the accuracy of the calculated tryptophan intake, it is almost impossible to 100% accurately determine dietary intake without more extensive information on the type and frequency of food consumed. The amount and detail of the information needed makes it impossible to obtain in practice with the current means available. It should also be noted that the use of the USDA food database is a limitation, since American food is not the same as Spanish food. However, a similar database for Spanish food is not currently available. Therefore, given that all calculations were made using the method considered best practice for estimating tryptophan intake, any deviations, positive or negative, from the real value would apply to the whole sample and relative intakes should remain the same and allow for accurate comparison between the intake quartiles. One data point that could be easily included, however, is the potential use of tryptophan supplements by the students which may affect their total daily intake. This information could be incorporated into the questionnaire administered to the students in future data collection rounds within the uniHcos Project. 

A significant limitation of the study is the presence of confounding variables not considered in the analyses. Some of these confounders which could significantly impact sleep include coffee and alcohol consumption, which could be significant given that the subjects are undergraduate students. Also, the intake of other nutrients, such as LNAAs, omega-3, and fiber, or the overall quality of the diet can be important for sleep quality. Finally, the possible influence of physical activity on sleep has also not been included as a confounder.

One of the most important limitations of the study is the nature of sleep itself. The sleep–wake cycle is a complex phenomenon. Many factors can disturb sleep in university students: diet, lifestyle (smoking, alcohol and drug use, exercise, etc.), environment, health, stress, travel, and numerous other physical, psychological, biological, and social factors. The factors that influence sleep are vast and it is thought that some still remain unidentified. Given this, to determine the effect that a single factor, such as tryptophan intake, has on sleep more accurately, it is necessary to adjust for the other possible confounders present in the studied population. In this study, however, our ability to do this is very limited due to the constraints of the data available. However, in future studies, information on confounding factors should be considered and the results adjusted for them.

## 6. Conclusions

Our results demonstrate the potential benefits of tryptophan intake for sleep improvement. Furthermore, sleep duration partially mediated this association. The findings from this extensive study support the potential benefit of tryptophan intake in the prevention and management of sleep issues, likely due to its positive effects on sleep quality. Since the underlying mechanisms of these phenomena are not fully understood, additional mechanistic studies investigating the impact of tryptophan consumption on sleep are warranted.

In conclusion, an association was found between a lower consumption of tryptophan and worse sleep in Spanish university students. Future studies should be carried out considering the large sample size, gender bias, other age groups, and that other social factors may influence sleep quality.

## Figures and Tables

**Table 1:** Sociodemographic characteristics of the university students.

	Total N = 11,485 (100%)	Male n = 3138 (27.3%)	Female n = 8347 (72.7%)	*p*
**Age** (Median ± SD)	20.11 ± 4.62	20.47 ± 5.17	19.97 ± 4.38	<0.001
**Housing**				<0.001
Family home	5213 (45.4%)	1537 (49.0%)	3676 (44.0%)	<0.001
University residence	1303 (11.3%)	327(10.4%)	976 (11.7%)	0.050
Own home	307(2.7%)	112 (3.6%)	195 (2.3%)	<0.001
Other	152(1.3%)	31 (1.0%)	121 (1.4%)	0.091
Rental	4510 (39.3%)	1131 (36.0%)	3379 (40.5%)	<0.001
**Coexistence**				<0.001
Parents	5253 (45.7%)	1515 (48.3%)	3738 (44.8%)	<0.001
Roommates	4627 (40.3%)	1166 (37.2%)	3461 (41.5%)	<0.001
Partner	60.4 (5.3%)	146 (4.7%)	458 (5.5%)	0.088
Alone	1001 (8.7%)	311 (9.9%)	690 (8.3%)	0.007
**Marital status**				0.140
Single	10,461 (91.1%)	2843 (90.6%)	7618 (91.3%)	0.240
Married	1024 (8.9%)	295 (9.4%)	729 (8.7%)	0.240
**Employment status**				0.007
Unemployed	10,261 (89.3%)	2764 (88.1%)	7487 (89.7%)	0.014
Employed	1234 (10.7%)	374 (11.9%)	860 (10.3%)	0.014
**Specialization**				<0.001
Humanity	1387 (12.1%)	297 (9.5%)	1090 (13.1%)	<0.001
Science College	1741 (15.2%)	571 (18.2%)	1170 (14.0%)	<0.001
Medical College	2537 (22.1%)	564 (18.0%)	1973 (23.7%)	<0.001
Management	4656 (40.6%)	1005 (32.1%)	3651 (43.8%)	<0.001
Engineering and architecture	1155 (10.1%)	698 (22.3%)	457 (5.5%)	<0.001

**Table 2:** (**a**) Self-reported sleep problems. (**b**) Insomnia-related (self-reported and Athens Insomnia Scale) sleep problems.

(a)				
	Total N = 11,485 (100%)	Male n = 3138 (27.3%)	Female n = 8347 (72.7%)	*p*
**Sleep duration** (Median ± SD) (h/day)	7.49 ± 1.24	7.44 ± 1.19	7.51 ± 1.25	0.007
**Short sleep duration** (<7 h/day)	5860 (51.0%)	1643 (52.4%)	4217 (50.5%)	0.041
**Sleep Efficiency** (Inadequate)	6315 (55.0%)	1967 (62.7%)	4348 (52.1%)	<0.001
**Difficulty to sleep**				<0.001
Never	2333 (20.3%)	754 (24.0%)	1579 (18.9%)	<0.001
Some days	4633 (40.3%)	1362 (43.4%)	3271 (39.2%)	<0.001
Several days	2556 (22.3%)	591(18.8%)	1965 (23.5%)	<0.001
Most days	1383 (12.0%)	297 (9.5%)	1086 (13.0%)	<0.001
Every day	580 (5.1%)	134 (4.3%)	446 (5.3%)	0.029
**Wake up several times while sleeping**				<0.001
Never	2636 (23.0%)	955 (30.4%)	1681 (20.1%)	<0.001
Some days	4600 (40.1%)	1308 (41.7%)	3292 (39.4%)	0.025
Several days	2345 (20.4%)	514 (16.4%)	1831 (21.9%)	<0.001
Most days	1360 (11.8%)	247 (7.9%)	1113 (13.3%)	<0.001
Every day	544 (4.7%)	114 (3.6%)	430 (5.2%)	<0.001
**Wake up early**				<0.001
Never	3784 (32.9%)	1106 (35.2%)	2678 (32.1%)	0.002
Some days	4407 (38.4%)	1281 (40.8%)	3126 (37.5%)	0.001
Several days	1735 (15.1%)	442 (14.1%)	1293 (15.5%)	0.062
Most days	1090 (9.5%)	220 (7.0%)	870 (10.4%)	<0.001
Every day	469 (4.1%)	89 (2.8%)	380 (4.6%)	<0.001
(**b**)				
	**Total** **N = 1380 (100%)**	**Male** **n = 334 (24.2%)**	**Female** **n = 1046 (75.8%)**	
**Insomnia**	777 (56.3%)	154 (46.1%)	623 (59.6%)	<0.001
**Athens Insomnia Scale**				
**Sleep induction**				0.079
No problem	441 (32.0%)	102 (30.5%)	339 (32.4%)	0.517
Slightly delayed	517 (37.5%)	144 (43.1%)	373 (35.7%)	0.015
Markedly delayed	348 (25.2%)	74 (22.2%)	274 (26.2%)	0.143
Very delayed or did not sleep at all	74 (5.4%)	14 (4.2%)	60 (5.7%)	0.289
**Wake up in the night**				0.323
No problem	568 (41.2%)	146 (43.7%)	422 (40.3%)	0.272
Slightly delayed	537 (38.9%)	133 (39.8%)	404 (39.6%)	0.948
Markedly delayed	223 (16.2%)	44 (13.2%)	179 (17.1%)	0.092
Very delayed or did not sleep at all	52 (3.8%)	11 (3.3%)	41 (3.9%)	0.616
**Wake up early end**				0.339
No problem	587 (43.5%)	151 (45.2%)	436 (41.7%)	0.260
Slightly delayed	580 (42.0%)	140 (41.9%)	440 (42.1%)	0.949
Markedly delayed	189 (13.7%)	37 (11.1%)	152 (14.5%)	0.116
Very delayed or did not sleep at all	24 (1.7%)	6 (1.8%)	18 (1.7%)	0.903
**Total sleep duration**				0.218
No problem	509 (36.9%)	139 (41.6%)	370 (35.4%)	0.041
Slightly delayed	590 (428%)	133 (39.8%)	457 (43.7%)	0.210
Markedly delayed	249 (18.0%)	54 (16.2%)	195 (18.6%)	0.321
Very delayed or did not sleep at all	32 (2.3%)	8 (2.4%)	24 (2.3%)	0.916
**Sleep quality**				0.055
No problem	532 (38.6%)	148 (44.3%)	384 (36.7%)	0.013
Slightly delayed	555 (40.2%)	128 (38.3%)	427 (40.8%)	0.417
Markedly delayed	245 (17.8%)	47 (14.1%)	198 (18.9%)	0.046
Very delayed or did not sleep at all	48 (3.5%)	11 (3.3%)	37 (3.5%)	0.862
**Wellbeing in the day**				<0.001
No problem	575 (41.7%)	175 (52.4%)	400 (38.2%)	<0.001
Slightly delayed	560 (40.6%)	119 (35.6%)	441 (42.2%)	0.033
Markedly delayed	187 (13.6%)	35 (10.5%)	152 (14.5%)	0.063
Very delayed or did not sleep at all	58 (4.2%)	5 (1.5%)	53 (5.1%)	0.004
**Daytime operation**				<0.001
No problem	663 (48.0%)	202 (60.5%)	461 (44.1%)	<0.001
Slightly delayed	488 (35.4%)	101 (30.2%)	387 (37.0%)	0.024
Markedly delayed	182 (13.2%)	26 (7.8%)	156 (14.9%)	<0.001
Very delayed or did not sleep at all	47 (3.4%)	5 (1.5%)	42 (4.0%)	0.028
**Daytime sleepiness**				<0.001
No problem	394 (28.6%)	135 (40.4%)	259 (24.8%)	<0.001
Slightly delayed	723 (52.4%)	161 (48.2%)	562 (53.7%)	0.078
Markedly delayed	213 (15.4%)	33 (9.9%)	180 (17.2%)	0.001
Very delayed or did not sleep at all	50 (3.6%)	5 (1.5%)	45 (4.3%)	0.017
**Total sleep**				
Total score (Median ± SD)	6.79 ± 4.48	5.85 ± 4.10	7.10 ± 4.55	<0.001
0–3: No insomnia	366 (28.5%)	257 (24.6%)	109 (32.6%)	0.006
4–7: Subclinical insomnia	492 (35.7%)	361 (34.5%)	131 (39.2%)	0.124
8–14: Clinically significant insomnia	427 (30.9%)	348 (33.3%)	79 (23.7%)	<0.001
15–24: Severe insomnia	95 (6.9%)	80 (7.6%)	15 (4.5%)	0.027
0–7 No insomnia	858 (62.2%)	240 (71.9%)	618 (59.1%)	<0.001
8–24 Insomnia	522 (37.8%)	94 (28.1%)	428 (40.9%)	<0.001

**Table 3:** Tryptophan requirements and intakes.

	Total N = 11,485 (100%)	Male n = 3138 (27.3%)	Female n = 8347 (72.7%)	*p*
**Tryptophan requirement ^a^** (Median ± SD) (mg/day)	285.11 ± 57.58	332.57 ± 57.03	268.03 ± 47.66	<0.001
**Tryptophan intake**				
Median ± SD (mg/day)	692.16 ± 246.61	731.84 ± 246.86	677.24 ± 244.87	<0.001
Q1(<526.43 mg/day)	2866 (25.0%)	595 (19.0%)	2271 (27.2%)	<0.001
Q2 (526.43–663.65 mg/day)	2860 (24.9%)	782 (24.9%)	2078 (24.9%)	1
Q3 (663.65–841.60 mg/day)	2884 (25.1%)	847 (27.0%)	2037 (24.4%)	0.004
Q4 (>841.60 mg/day)	2875 (25.0%)	914 (29.1%)	1961 (23.5%)	<0.001
**Intake/Requirement ratio**	2.51 ± 1.01	2.27 ± 0.85	2.60 ± 1.05	<0.001

^a^ 4.5 mg/kg bodyweight/day.

**Table 4:** Risk of sleep-related problems by tryptophan consumption quartile.

	Sleep Duration (<7 h/day)			Sleep Efficiency (Adequate)			Insomnia (Yes)			Athens Insomnia Scale (Insomnia)		
	ORc	95% CI		ORc	95% CI		ORc	95% CI		ORc	95% CI	
**Male**												
Q1	1.26	1.02	1.55	0.88	0.72	1.10	1.48	0.84	2.61	2.56	1.36	4.82
Q2	1.00	0.83	1.21	0.83	0.68	1.04	1.35	0.74	2.47	1.39	0.68	2.79
Q3	1.05	0.87	1.27	0.92	0.76	1.12	1.00	0.54	1.86	1.23	0.60	2.54
Q4	1 Reference			1 Reference			1 Reference			1 Reference		
**Female**												
Q1	1.19	1.05	1.34	0.93	0.82	1.05	1.48	1.06	2.06	1.47	1.10	2.05
Q2	0.95	0.84	1.08	1.00	0.87	1.12	1.04	0.73	1.49	1.22	0.85	1.77
Q3	1.12	1.00	1.27	0.98	0.87	1.11	1.22	0.83	1.80	1.23	0.83	1.83
Q4	1 Reference			1 Reference			1 Reference			1 Reference		
**Total**												
Q1	1.19	1.07	1.34	0.87	0.78	0.97	1.57	1.18	2.08	1.74	1.30	2.33
Q2	0.96	0.87	1.08	0.93	0.83	1.03	1.15	0.49	1.56	1.32	0.95	1.82
Q3	1.10	1.00	1.27	0.96	0.86	1.06	1.18	0.85	1.63	1.26	0.89	1.77
Q4	1 Reference			1 Reference			1 Reference			1 Reference		

The darkest green represents the lowest values of the ORcs and the darkest reds the highest values with the yellows and browns in between.

**Table 5:** Risk of insomnia according to the Athens Insomnia Scale by tryptophan consumption quartile and academic specialization.

	Humanity n = 1387 (12.1%)			Science College n = 1741 (15.2%)			Medical College n = 2537 (22.1%)			Management n = 4656 (40.6%)			Engineering and Architecture n = 1155 (10.1%)		
	ORc	95% CI		ORc	95% CI		ORc	95% CI		ORc	95% CI		ORc	95% CI	
Male															
Q1	3.00	0.39	23.07	2.10	0.56	7.81	-	-	-	1.48	0.49	4.55	2.78	0.70	11.10
Q2	4.80	0.65	35.19	0.67	0.11	4.19	-	-	-	1.25	0.37	4.32	0.30	0.04	1.68
Q3	0.86	0.06	11.25	0.67	0.08	2.80	-	-	-	1.00	0.31	3.18	1.73	0.34	8.87
Q4	1 Reference			1 Reference			1 Reference			1 Reference			1 Reference		
Female															
Q1	3.15	1.04	9.55	1.46	0.62	3.49	1.35	0.63	2.89	1.49	0.95	2.42	0.53	0.13	2.20
Q2	3.41	1.01	11.5	1.13	0.41	30.9	1.00	0.44	2.12	1.21	072	2.05	1.50	0.20	11.08
Q3	3.30	0.96	11.57	0.92	0.32	2.63	1.01	0.42	2.43	1.27	0.72	2.26	1.00	0.19	6.25
Q4	1 Reference			1 Reference			1 Reference			1 Reference			1 Reference		
Total															
Q1	3.50	1.29	8.68	1.64	0.97	3.39	2.04	1.02	4.11	1.57	1.02	2.44	1.28	0.49	3.37
Q2	3.81	1.35	10.75	1.04	0.437	2.47	1.53	0.74	3.15	1.27	0.79	2.06	0.50	0.15	1.53
Q3	2.69	1.35	10.75	0.79	0.33	1.91	1.50	0.67	3.34	1.19	0.72	1.98	1.36	0.40	4.50
Q4	1 Reference			1 Reference			1 Reference			1 Reference			1 Reference		

The darkest green represents the lowest values of the ORcs and the darkest reds the highest values with the yellows and browns in between.

## Data Availability

The datasets analyzed in the current study are available from the corresponding author upon reasonable request.

## References

[1] Spiegel K., Tasali E., Leproult R., Van Cauter E. (2009). Effects of poor and short sleep on glucose metabolism and obesity risk. Nat. Rev. Endocrinol..

[2] Alafif N. (2024). Association between consumption of tryptophan with sleep quality in King Saud University students. J. King Saud Univ.-Sci..

[3] Christensen D.S., Zachariae R., Amidi A., Wu L.M. (2022). Sleep and allostatic load: A systematic review and meta-analysis. Sleep Med. Rev..

[4] Calhoun D.A., Harding S.M. (2010). Sleep and hypertension. Chest.

[5] Ramar K., Malhotra R.K., Carden K.A., Martin J.L., Abbasi-Feinberg F., Aurora R.N., Kapur V.K., Olson E.J., Rosen C.L., Rowley J.A. (2021). Sleep is essential to health: An American Academy of Sleep Medicine position statement. J. Clin. Sleep Med..

[6] de Entrambasaguas M., Romero O., Guevara J.A.C., de Larrinaga A.Á.R., Cañellas F., Salud J.P., Díaz H.P. (2023). The prevalence of insomnia in Spain: A stepwise addition of ICSD-3 diagnostic criteria and notes. Sleep Epidemiol..

[7] Carrión-Pantoja S., Prados G., Chouchou F., Holguín M., Mendoza-Vinces Á., Expósito-Ruiz M., Fernández-Puerta L. (2022). Insomnia symptoms, sleep hygiene, mental health, and academic performance in Spanish university students: A cross-sectional study. J. Clin. Med..

[8] AlDabal L., BaHammam A.S. (2011). Metabolic, endocrine, and immune consequences of sleep deprivation. Open Respir. Med. J..

[9] Vélez J.C., Souza A., Traslaviña S., Barbosa C., Wosu A., Andrade A., Frye M., Fitzpatrick A.L., Gelaye B., Williams M.A. (2013). The epidemiology of sleep quality and consumption of stimulant beverages among Patagonian Chilean college students. Sleep Disord..

[10] Edwards M.K., Loprinzi P.D. (2017). Experimentally increasing sedentary behavior results in decreased sleep quality among young adults. Ment. Health Phys. Act..

[11] Gilbert S.P., Weaver C.C. (2010). Sleep quality and academic performance in university students: A wake-up call for college psychologists. J. Coll. Stud. Psychother..

[12] Hershner S.D., Chervin R.D. (2014). Causes and consequences of sleepiness among college students. Nat. Sci. Sleep.

[13] Brown F.C., Buboltz W.C., Soper B. (2002). Relationship of sleep hygiene awareness, sleep hygiene practices, and sleep quality in university students. Behav. Med..

[14] Kloss J.D., Nash C.O., Horsey S.E., Taylor D.J. (2011). The delivery of behavioral sleep medicine to college students. J. Adolesc. Health.

[15] Forquer L.M., Camden A.E., Gabriau K.M., Johnson C.M. (2008). Sleep patterns of college students at a public university. J. Am. Coll. Health.

[16] Dietrich S.K., Francis-Jimenez C.M., Knibbs M.D., Umali I.L., Truglio-Londrigan M. (2016). Effectiveness of sleep education programs to improve sleep hygiene and/or sleep quality in college students: A systematic review. JBI Evid. Synth..

[17] Wolfson A.R. (2010). Adolescents and emerging adults’ sleep patterns: New developments. J. Adolesc. Health.

[18] Altun İ., Cınar N., Dede C. (2012). The contributing factors to poor sleep experiences in according to the university students: A cross-sectional study. J. Res. Med. Sci. Off. J. Isfahan Univ. Med. Sci..

[19] Friedrich A., Schlarb A.A. (2018). Let’s talk about sleep: A systematic review of psychological interventions to improve sleep in college students. J. Sleep Res..

[20] Vail-Smith K., Felts W.M., Becker C. (2009). Relationship between sleep quality and health risk behaviors in undergraduate college students. Coll. Stud. J..

[21] Taylor D.J., Gardner C.E., Bramoweth A.D., Williams J.M., Roane B.M., Grieser E.A., Tatum J.I. (2011). Insomnia and mental health in college students. Behav. Sleep Med..

[22] Schlarb A.A., Kulessa D., Gulewitsch M.D. (2012). Sleep characteristics, sleep problems, and associations of self-efficacy among German university students. Nat. Sci. Sleep.

[23] Nadorff M.R., Nazem S., Fiske A. (2011). Insomnia symptoms, nightmares, and suicidal ideation in a college student sample. Sleep.

[24] Medeiros A.L.D., Mendes D.B., Lima P.F., Araujo J.F. (2001). The relationships between sleep-wake cycle and academic performance in medical students. Biol. Rhythm Res..

[25] Trockel M.T., Barnes M.D., Egget D.L. (2000). Health-related variables and academic performance among first-year college students: Implications for sleep and other behaviors. J. Am. Coll. Health.

[26] Lund H.G., Reider B.D., Whiting A.B., Prichard J.R. (2010). Sleep patterns and predictors of disturbed sleep in a large population of college students. J. Adolesc. Health.

[27] Kelly W.E., Kelly K.E., Clanton R.C. (2001). The relationship between sleep length and grade-point average among college students. Coll. Stud. J..

[28] Gaultney J.F. (2010). The prevalence of sleep disorders in college students: Impact on academic performance. J. Am. Coll. Health.

[29] Spielman A.J., Caruso L.S., Glovinsky P.B. (1987). A behavioral perspective on insomnia treatment. Psychiatr. Clin. N. Am..

[30] Karna B., Sankari A., Tatikonda G. (2024). Sleep Disorder.

[31] St-Onge M., Mikic A., Pietrolungo C.E. (2016). Effects of diet on sleep quality. Adv. Nutr..

[32] Godos J., Grosso G., Castellano S., Galvano F., Caraci F., Ferri R. (2021). Association between diet and sleep quality: A systematic review. Sleep Med. Rev..

[33] Peuhkuri K., Sihvola N., Korpela R. (2012). Diet promotes sleep duration and quality. Nutr. Res..

[34] Knowlden A.P., Hackman C.L., Sharma M. (2016). Systematic review of dietary interventions targeting sleep behavior. J. Altern. Complement. Med..

[35] Sutanto C.N., Wang M.X., Tan D., Kim J.E. (2020). Association of sleep quality and macronutrient distribution: A systematic review and meta-regression. Nutrients.

[36] Zuraikat F.M., Wood R.A., Barragán R., St-Onge M. (2021). Sleep and diet: Mounting evidence of a cyclical relationship. Annu. Rev. Nutr..

[37] Sutanto C.N., Loh W.W., Kim J.E. (2022). The impact of tryptophan supplementation on sleep quality: A systematic review, meta-analysis, and meta-regression. Nutr. Rev..

[38] Silber B.Y., Schmitt J. (2010). Effects of tryptophan loading on human cognition, mood, and sleep. Neurosci. Biobehav. Rev..

[39] EFSA Panel on Dietetic Products, Nutrition and Allergies (NDA) (2012). Scientific opinion on dietary reference values for protein. EFSA J..

[40] Institute of Medicine (US) Standing Committee on the Scientific Evaluation of Dietary Reference Intakes (2005). Dietary Reference Intakes for Energy, Carbohydrate, Fiber, Fat, Fatty Acids, Cholesterol, Protein, and Amino Acids.

[41] Forbes G.B. (1974). Joint FAO/WHO ad hoc Expert Committee, Energy and Protein Requirements.

[42] World Health Organization (1985). Energy and protein requirements: Report of a joint FAO/WHO/UNU expert consultation. Energy and Protein Requirements: Report of a Joint FAO/WHO/UNU Expert Consultation.

[43] Moore P., Landolt H., Seifritz E., Clark C., Bhatti T., Kelsoe J., Rapaport M., Gillin J.C. (2000). Clinical and physiological consequences of rapid tryptophan depletion. Neuropsychopharmacology.

[44] Kitano N., Tsunoda K., Tsuji T., Osuka Y., Jindo T., Tanaka K., Okura T. (2014). Association between difficulty initiating sleep in older adults and the combination of leisure-time physical activity and consumption of milk and milk products: A cross-sectional study. BMC Geriatr..

[45] Nisar M., Mohammad R.M., Arshad A., Hashmi I., Yousuf S.M., Baig S., Hashmi S.M.I. (2019). Influence of dietary intake on sleeping patterns of medical students. Cureus.

[46] Yasuda J., Yoshizaki T., Yamamoto K., Yoshino M., Ota M., Kawahara T., Kamei A. (2019). Association of frequency of milk or dairy product consumption with subjective sleep quality during training periods in Japanese elite athletes: A cross-sectional study. J. Nutr. Sci. Vitaminol..

[47] Bravo R., Matito S., Cubero J., Paredes S.D., Franco L., Rivero M., Rodríguez A.B., Barriga C. (2013). Tryptophan-enriched cereal intake improves nocturnal sleep, melatonin, serotonin, and total antioxidant capacity levels and mood in elderly humans. Age.

[48] Mohajeri M.H., Wittwer J., Vargas K., Hogan E., Holmes A., Rogers P.J., Goralczyk R., Gibson E.L. (2015). Chronic treatment with a tryptophan-rich protein hydrolysate improves emotional processing, mental energy levels and reaction time in middle-aged women. Br. J. Nutr..

[49] Alkhatatbeh M.J., Khwaileh H.N., Abdul-Razzak K.K. (2021). High prevalence of low dairy calcium intake and association with insomnia, anxiety, depression and musculoskeletal pain in university students from Jordan. Public Health Nutr..

[50] Meier T.B., Savitz J. (2022). The kynurenine pathway in traumatic brain injury: Implications for psychiatric outcomes. Biol. Psychiatry.

[51] Platten M., Nollen E.A., Röhrig U.F., Fallarino F., Opitz C.A. (2019). Tryptophan metabolism as a common therapeutic target in cancer, neurodegeneration and beyond. Nat. Rev. Drug Discov..

[52] Clarke G., Grenham S., Scully P., Fitzgerald P., Moloney R.D., Shanahan F., Dinan T.G., Cryan J. (2013). The microbiome-gut-brain axis during early life regulates the hippocampal serotonergic system in a sex-dependent manner. Mol. Psychiatry.

[53] Fernández Villa T., Alguacil Ojeda J., Ayán Pérez C., Bueno Cavanillas A., Cancela Carral J.M., Capelo Álvarez R., Delgado Rodríguez M., Jiménez Mejías E., Jiménez Moleón J.J., Llorca Díaz J. (2013). Proyecto UNIHCOS: Cohorte dinámica de estudiantes universitarios para el estudio del consumo de drogas y otras adicciones. Rev. Española Salud Pública.

[54] Andréu M.M., de Larrinaga A.Á.R., Pérez J.A., Martínez M.Á.M., Cuesta F.J.P., Guerra A.J.A., Santo-Tomás O.R., Luque M.J.J., Isern F.J.S., Sanz T.C. (2016). Sueño saludable: Evidencias y guías de actuación. Documento oficial de la Sociedad Española de Sueño. Rev. Neurol..

[55] Gómez-Benito J., Ruiz C., Guilera G. (2011). A Spanish version of the Athens Insomnia Scale. Qual. Life Res..

[56] Soldatos C.R., Dikeos D.G., Paparrigopoulos T.J. (2000). Athens Insomnia Scale: Validation of an instrument based on ICD-10 criteria. J. Psychosom. Res..

[57] Chung K., Yeung W., Ho F.Y., Yung K., Yu Y., Kwok C. (2015). Cross-cultural and comparative epidemiology of insomnia: The Diagnostic and statistical manual (DSM), International classification of diseases (ICD) and International classification of sleep disorders (ICSD). Sleep Med..

[58] Cao X., Wang S., Zhong B., Zhang L., Ungvari G.S., Ng C.H., Li L., Chiu H.F., Lok G.K., Lu J. (2017). The prevalence of insomnia in the general population in China: A meta-analysis. PLoS ONE.

[59] Riera-Sampol A., Rodas L., Martínez S., Moir H.J., Tauler P. (2022). Caffeine intake among undergraduate students: Sex differences, sources, motivations, and associations with smoking status and self-reported sleep quality. Nutrients.

[60] Gallego-Gómez J.I., González-Moro M.T.R., González-Moro J.M.R., Vera-Catalán T., Balanza S., Simonelli-Muñoz A.J., Rivera-Caravaca J.M. (2021). Relationship between sleep habits and academic performance in university Nursing students. BMC Nurs..

[61] Suardiaz-Muro M., Ortega-Moreno M., Morante-Ruiz M., Monroy M., Ruiz M.A., Martín-Plasencia P., Vela-Bueno A. (2023). Sleep quality and sleep deprivation: Relationship with academic performance in university students during examination period. Sleep Biol. Rhythm..

[62] Navarro-Martínez R., Chover-Sierra E., Colomer-Pérez N., Vlachou E., Andriuseviciene V., Cauli O. (2020). Sleep quality and its association with substance abuse among university students. Clin. Neurol. Neurosurg..

[63] Lieberman H.R., Agarwal S., Fulgoni V.L. (2016). Tryptophan intake in the US adult population is not related to liver or kidney function but is associated with depression and sleep outcomes. J. Nutr..

[64] Razeghi Jahromi S., Togha M., Ghorbani Z., Hekmatdoost A., Khorsha F., Rafiee P., Shirani P., Nourmohammadi M., Ansari H. (2019). The association between dietary tryptophan intake and migraine. Neurol. Sci..

[65] Suga H., Asakura K., Kobayashi S., Nojima M., Sasaki S. (2018). Association between habitual tryptophan intake and depressive symptoms in young and middle-aged women. J. Affect. Disord..

[66] Sutanto C., Xia X., Heng C.W., Tan Y.S., Gan A.X., Wang X.F., Fam J., Kim J.E. (2022). Impact of 5-Hydroxytryptophan Supplementation on Gut Microbiota Composition of Older Adults with Different Sleep Status in Singapore: A Randomized Controlled Trial. Curr. Dev. Nutr..

[67] Sutanto C.N., Xia X., Heng C.W., Tan Y.S., Lee D.P.S., Fam J., Kim J.E. (2024). The impact of 5-hydroxytryptophan supplementation on sleep quality and gut microbiota composition in older adults: A randomized controlled trial. Clin. Nutr..

[68] da Silva I.T.S.A., da Silva Fidelis D.E., de Souza R.F., de Sousa Fernandes M.S. (2024). Comment on “The impact of 5-hydroxytryptophan supplementation on sleep quality and gut microbiota composition in older adults: A randomized controlled trial” clinical nutrition 2024. Clin. Nutr..

[69] van Egmond L., Tan X., Sjögren P., Cederholm T., Benedict C. (2019). Association between healthy dietary patterns and self-reported sleep disturbances in older men: The ULSAM study. Nutrients.

[70] Min C., Kim H.-J., Park I.-S., Park B., Kim J.-H., Sim S., Hyo G.C. (2018). The association between sleep duration, sleep quality, and food consumption in adolescents: A cross-sectional study using the Korea Youth Risk Behavior Web-based Survey. BMJ Open.

[71] Sato-Mito N., Sasaki S., Murakami K., Okubo H., Takahashi Y., Shibata S., Yamada K., Sato K., Freshmen in Dietetic Courses Study II group (2011). The midpoint of sleep is associated with dietary intake and dietary behavior among young Japanese women. Sleep Med..

[72] Thompson R.S., Roller R., Mika A., Greenwood B.N., Knight R., Chichlowski M., Berg B.M., Fleshner M. (2017). Dietary prebiotics and bioactive milk fractions improve NREM sleep, enhance REM sleep rebound and attenuate the stress-induced decrease in diurnal temperature and gut microbial alpha diversity. Front. Behav. Neurosci..

[73] Komada Y., Okajima I., Kuwata T. (2020). The Effects of Milk and Dairy Products on Sleep: A Systematic Review. Int. J. Environ. Res. Public Health.

[74] Cavero-Redondo I., Alvarez-Bueno C., Sotos-Prieto M., Gil A., Martinez-Vizcaino V., Ruiz J.R. (2019). Milk and Dairy Product Consumption and Risk of Mortality: An Overview of Systematic Reviews and Meta-Analyses. Adv. Nutr..

[75] Heine W., Radke M., Wutzke K.-D. (1995). The significance of tryptophan in human nutrition. Amino Acids.

[76] Peters J.C. (1991). Tryptophan nutrition and metabolism: An overview. Kynurenine and Serotonin Pathways: Progress in Tryptophan Research.

[77] Ardiansyah S., Shirakawa H., Inagawa Y., Koseki T., Komai M. (2011). Regulation of blood pressure and glucose metabolism induced by L-tryptophan in stroke-prone spontaneously hypertensive rats. Nutr. Metab..

[78] Fernstrom J.D. (2016). A perspective on the safety of supplemental tryptophan based on its metabolic fates. J. Nutr..

[79] Fernstrom J.D. (1983). Role of precursor availability in control of monoamine biosynthesis in brain. Physiol. Rev..

[80] Muldoon M.F., Mackey R.H., Williams K.V., Korytkowski M.T., Flory J.D., Manuck S.B. (2004). Low central nervous system serotonergic responsivity is associated with the metabolic syndrome and physical inactivity. J. Clin. Endocrinol. Metab..

[81] Zimmermann R.C., McDougle C.J., Schumacher M., Olcese J., Mason J.W., Heninger G.R., Price L.H. (1993). Effects of acute tryptophan depletion on nocturnal melatonin secretion in humans. J. Clin. Endocrinol. Metab..

[82] Dawson D., Encel N. (1993). Melatonin and sleep in humans. J. Pineal Res..

[83] Smith W.G. Does Gender Influence Online Survey Participation? A Record-Linkage Analysis of University Faculty Online Survey Response Behavior; Online Submission. https://eric.ed.gov/?id=ED501717.

